# Efficacy of stem cell-derived extracellular vesicles in animal models of autism spectrum disorder: a systematic review and meta-analysis

**DOI:** 10.3389/fpsyt.2026.1956214

**Published:** 2026-10-12

**Authors:** Zhen Zheng, Manfen Ji, Yayong Liang, Sinian Pan

**Affiliations:** Department of Pediatrics, The Third Affiliated Hospital, Sun Yat-sen University, Guangzhou, China

**Keywords:** animal models, autism spectrum disorder, meta-analysis, neuroinflammation, stem cell-derived extracellular vesicles

## Abstract

**Background:**

Autism spectrum disorder (ASD) is a complicated neurodevelopmental condition that currently lacks a targeted therapy. Although stem cell-derived extracellular vesicles (EVs) represent a potential therapeutic approach for neurological disorders, their efficacy in ASD remains unclear. The objective of this study was to investigate the efficacy of stem cell-derived EVs on autistic-like behaviors in animal models of ASD and to explore the underlying mechanisms.

**Methods:**

Relevant studies published up to April 20, 2026 were searched across five databases. Data were pooled using Review Manager 5.4. Outcome measures included behavioral tests and inflammatory cytokine levels.

**Results:**

Out of 1,165 initially retrieved articles, five animal studies met the inclusion criteria. Pooled analyses demonstrated that EV treatment improved autistic-like behaviors in the self-grooming test, three-chamber social interaction test, and marble burying test, and decreased levels of proinflammatory cytokines IL-6 and IL-1β (all *P* < 0.05). No notable enhancements were observed for the open field test, Morris Water Maze, TNF-α or IL-10 (all *P* > 0.05).

**Conclusion:**

Stem cell-derived EV therapy may improve repetitive behaviors and social deficits in ASD animal models, with observed associations with reduced IL-6 and IL-1β levels. These preclinical findings suggest promising translational potential of EV-based therapy for ASD.

**Systematic Review Registration:**

https://osf.io/c78xm, identifier 10.17605/OSF.IO/C78XM.

## 1 Introduction

Autism spectrum disorder (ASD) is a heterogeneous neurodevelopmental condition marked by impairments in social communication, narrow interests, and repetitive behaviors. The global prevalence of ASD has been rising steadily, placing a heavy burden on patients’ families and the whole society (1). Despite intensive research, the exact pathogenesis of ASD remains elusive. Current interventions mainly focus on behavioral and educational interventions (2). No specific targeted treatment is available to cure the core symptoms of ASD. Therefore, there is an urgent demand for new therapeutic strategies targeting the fundamental pathological mechanisms.

Stem cell-derived extracellular vesicles (EVs) have recently shown therapeutic potential in treating various neurological disorders (3–8). EVs contain abundant bioactive molecules, including mRNA, microRNA, DNA, and proteins (9). Compared with stem cell-based therapy, stem cell-derived EVs offer several benefits, including low immunogenicity, blood-brain barrier penetration, and simpler storage and operation. Stem cell-derived EVs exert protective effects through anti-inflammatory, anti-oxidative, anti-apoptotic, and neurotrophic mechanisms (10–14).

Recent findings indicate that neuroinflammation contributes significantly to the pathogenesis of ASD (15–17). Increased concentrations of pro-inflammatory cytokines, including interleukin-6 (IL-6) and interleukin-1β (IL-1β), have been reported in both individuals with ASD and animal models (15–17). Preclinical studies have reported that stem cell-derived EVs can improve behavioral deficits and attenuate neuroinflammation in animal models of ASD (18–21). However, these studies vary in study design, animal model types, stem cell sources, and intervention protocols, leading to inconclusive evidence.

To date, no systematic review or meta-analysis has summarized preclinical evidence of stem cell-derived EV therapy for ASD or elucidated its therapeutic mechanisms. Thus, we performed the first systematic review and meta-analysis of preclinical animal studies to determine the efficacy of stem cell-derived EVs on autistic-like behaviors and to explore the underlying mechanisms. Our study aimed to lay a foundation for the clinical translation of EV-based therapy for ASD.

## 2 Methods

### 2.1 Data sources and search strategy

We performed a systematic screening of literature published before April 20, 2026, in PubMed, Web of Science, Embase, Cochrane Library, and Scopus. We have retrospectively registered the protocol on the Open Science Framework (OSF; https://osf.io/c78xm). The search strategy employed a combination of terms associated with extracellular vesicles (e.g., “exosomes”, “exosome”, “extracellular vesicles”, “extracellular vesicle”, “vesicle”, “microparticle”, “secretory vesicles”, “exovesicles”, “exovesicle”, “endosomes”, “nanovesicle”) with terms related to autism spectrum disorder (e.g., “ASD”, “autism spectrum disorder”, “autistic disorder”, “autism”, “Asperger syndrome”, “pervasive developmental disorder”). Additionally, we reviewed the references of the relevant articles to uncover other eligible studies.

### 2.2 Inclusion and exclusion criteria

Studies meeting the following criteria were included: (1) conducted using animal models of ASD; (2) used stem cell-derived EVs as an intervention; (3) included a control group; (4) published in English; and (5) provided sufficient quantitative data for meta-analysis.

Studies meeting the following criteria were excluded: (1) lacking a control group; (2) duplicate literature; (3) reviews, conference abstracts, comments, or case reports; (4) clinical studies or *in vitro* experiments; and (5) incomplete data.

### 2.3 Study selection

Two investigators independently screened the titles and abstracts. Subsequently, the complete articles were assessed against the inclusion and exclusion criteria. If disagreement arose, a third investigator rendered the final judgment.

### 2.4 Data extraction

We obtained key data from eligible studies, including authors, publication year, author country, autism model, sources of stem cell-derived EVs, time of administration, route of administration, control group, dose of EVs, frequency, follow-up duration, and outcome assessments. When individual studies did not provide exact numerical results, we used WebPlotDigitizer to extract values from the published charts. Then we calculated the mean values and standard deviations (SDs). If a study only provided the standard error of the mean (SEM), we calculated the SD from the SEM using the equation SD = SEM × √n, where n stands for the sample size of the group.

### 2.5 Quality assessment

To evaluate the quality of studies, we applied the risk bias tool from the Systematic Review Centre for Laboratory Animal Experimentation (SYRCLE). Ten aspects were examined: random sequence generation, baseline characteristics, allocation concealment, random housing, blinding of participants and personnel, random outcome assessment, blinding of outcome assessment, incomplete outcome data, selective reporting, and other bias. Two independent reviewers assigned a rating of low, unclear, or high risk to each domain. Disagreements were resolved by consensus.

### 2.6 Outcome indicators

Primary outcome indicators: self-grooming test, three-chamber social interaction test, marble burying test, open field test, and Morris Water Maze.

Secondary outcome indicators: brain tissue levels of IL-6, IL-1β, TNF-α, and IL-10.

### 2.7 Statistical analysis

Review Manager 5.4 was used to perform data analysis. Between stem cell-derived EV treatment and control groups, the pooled effect size was derived as a standardized mean difference (SMD) with a 95% confidence interval (CI), and a forest plot was provided. Two-sided tests were employed, and *P* < 0.05 indicated statistical significance. Heterogeneity was quantified by I^2^. There was significant heterogeneity among the studies, including differences in animal species (mice vs. rats), ASD induction models (genetic vs. chemical/VPA), EV sources (bone marrow vs. umbilical cord), administration routes (intravenous vs. intranasal), and dose variations. Therefore, a random-effects model was adopted for all outcomes to uniformly account for the differences among the studies. For analyses involving at least three studies, sensitivity analysis was carried out through stepwise removal of individual studies. The SMD and 95% CI from the remaining studies were then compared with the overall estimate.

## 3 Results

### 3.1 Study selection process

Of the 1,165 articles found, 493 originated from PubMed, 263 from Web of Science, 394 from Embase, 9 from the Cochrane Library, 4 from Scopus, and 2 were retrieved from the reference lists of eligible studies. After excluding 396 duplicate articles, 769 articles remained for screening. After reviewing titles and abstracts, 644 articles were eliminated, and the remaining 125 articles underwent full-text evaluation. According to the established criteria, 120 articles were removed for the reasons below: 112 articles dealt with irrelevant topics, 4 articles lacked precise data of sample sizes, and 4 articles had no full text. Ultimately, 5 articles qualified for inclusion in this meta-analysis. **Figure 1** presents the PRISMA flow diagram.

**Figure 1:**
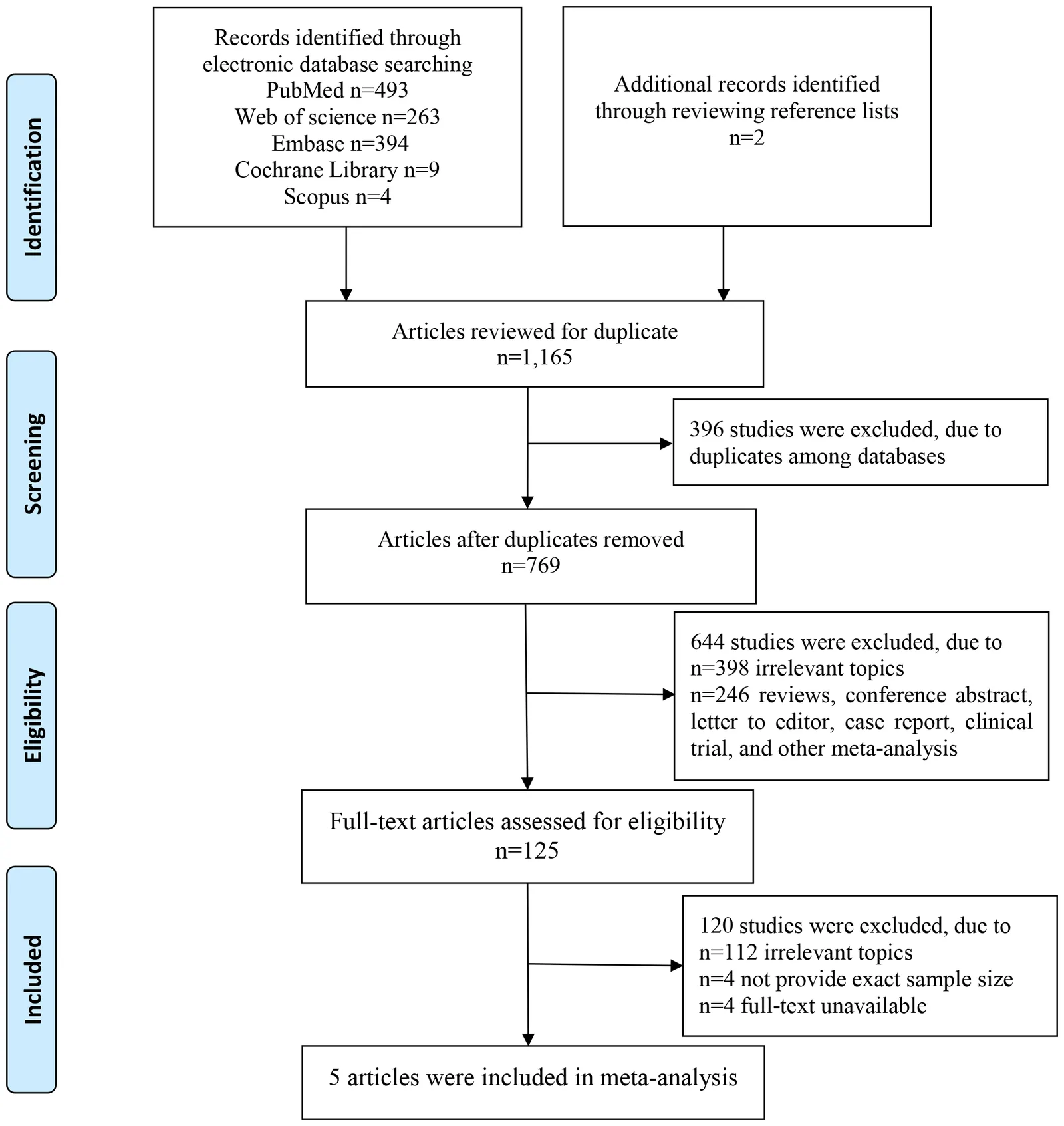
PRISMA flow diagram.

### 3.2 Study characteristics

**Table 1** presents the baseline characteristics of five included studies. Published from 2018 to 2025, the studies were performed in various countries, including Egypt, China, and Israel. In terms of animal models, one study used rats (18), three studies employed C57BL/6 mice (21–23), and one study utilized BTBR mice (24). Regarding the source of exosomes, two studies used bone marrow-derived mesenchymal stem cells (18, 24), and three studies applied umbilical cord mesenchymal stem cells (21–23). In terms of administration routes, one study administered exosomes via intravenous injection (18), and four studies administered intranasally (21–24). Regarding the dosage arrangement, three studies administered 100μg exosomes each time (18, 22, 23), one study administered 50μg exosomes each time (21), and another study did not specify the exact dosage of exosomes (24).

**Table 1:** Characteristics of the included studies.

First author, year	Country	Animals	Sources of extracellular vesicles	Time of administration	Route of administration	Control	Dose	Frequency	Follow-up	Outcomes
Elsherif, 2025	Egypt	Male Wistar rats	Bone marrow derived mesenchymal stem cells from white albino rat	3 weeks	Intravenous injection	Not mention	100μg	3	9 weeks	Three-chamber social interaction test, open field test, Morris Water Maze
Liang, 2020	China	Male C57BL/6 mice	Umbilical cord mesenchymal stem cells from human	6 weeks	Intranasal injection	Saline	100μg	Not mention	10–11 weeks	IL-6, IL-1β, TNF-α, IL-10
Perets, 2018	Israel	Male BTBR mice	Bone marrow derived mesenchymal stem cells from human	4 weeks	Intranasal injection	Saline	Not mention	6	Not mention	Self-grooming test
Qin, 2025	China	Male C57BL/6 mice	Umbilical cord mesenchymal stem cells from human	6–7 weeks	Intranasal injection	PBS	100μg	7	Not mention	Self-grooming test, three-chamber social interaction test, marble burying test, open field test
Wu, 2025	China	Male C57BL/6 mice	Umbilical cord mesenchymal stem cells from human	postnatal day 50	Intranasal injection	Not mention	50μg	4	11 weeks	Self-grooming test, three-chamber social interaction test, marble burying test, open field test, Morris Water Maze, IL-6, IL-1β, TNF-α, IL-10

### 3.3 Study quality

Risk of bias assessment was performed with the SYRCLE tool (**Figure 2**). Random sequence generation was unclear in all studies. Baseline characteristics of animals, such as age and gender, were generally reported. However, the influence of other factors still cannot be ruled out. Allocation concealment methods were not adequately described in any study. All studies reported random housing. Blinding of participants or personnel and randomization in outcome assessment were not described in any study. Blinding in outcome assessment was reported in only three studies (18, 21, 24). Other potential sources of bias remain uncertain due to insufficient information. In summary, the included studies showed unclear risk in multiple domains, and the overall bias risk was not judged as high.

**Figure 2:**
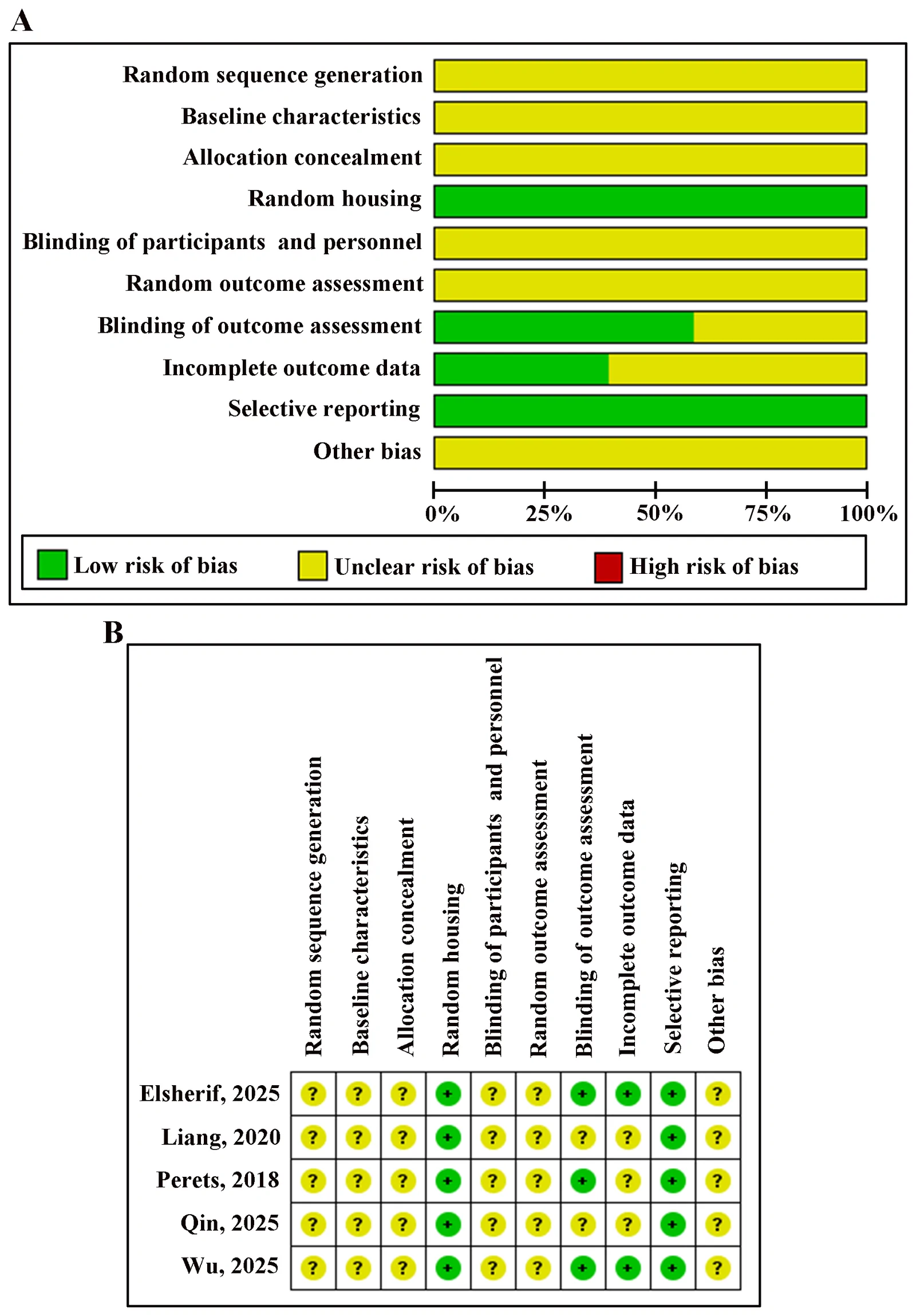
SYRCLE risk of bias assessment for included studies. **(A)** Risk of bias graph. **(B)** Risk of bias summary.

### 3.4 Stem cell-derived EV treatment ameliorated autistic-like behaviors in ASD animals

Multiple investigations have evaluated the effects of stem cell-derived EV treatment on autistic-like behaviors in ASD animals. Three studies (21, 23, 24) reported reduced self-grooming behavior in ASD animals subsequent to stem cell-derived EV treatment (SMD = -1.31, 95% CI: -1.88 to -0.74, I^2^ = 14%, *P* < 0.001) (**Figure 3A**). Sensitivity analysis revealed that omitting any single study did not affect the overall effect. Three studies (18, 21, 23) reported that after stem cell-derived EV treatment, the time spent with stranger 1 in the three-chamber social interaction test among ASD animals was increased (SMD = 2.40, 95% CI: 1.10 to 3.70, I^2^ = 69%, *P<* 0.001) (**Figure 3B**). Sensitivity analysis indicated that the overall finding was not dominated by any one study. Two studies (21, 23) assessed the effect of stem cell-derived EV intervention on the marble burying test, with significant reductions in the number of buried marbles observed in the ASD animals (SMD = -2.28, 95% CI: -4.17 to -0.40, I^2^ = 79%, *P* = 0.02) (**Figure 3C**). Three studies (18, 21, 23) reported that after stem cell-derived EV administration, there were no statistically significant variations in the time spent in the center of the open field test for ASD animals (SMD = 1.35, 95% CI: -1.28 to 3.98, I^2^ = 93%, *P* = 0.31) (**Figure 3D**). Sensitivity analysis revealed that omitting any single study did not affect the pooled effect. Two studies (18, 21) reported that after stem cell-derived EV treatment, ASD animals showed no statistically significant change in target quadrant time during the Morris Water Maze test (SMD = 0.92, 95% CI: -0.77 to 2.61, I^2^ = 84%, *P* = 0.29) (**Figure 3E**). An overview of these findings is presented in **Table 2**.

**Figure 3:**
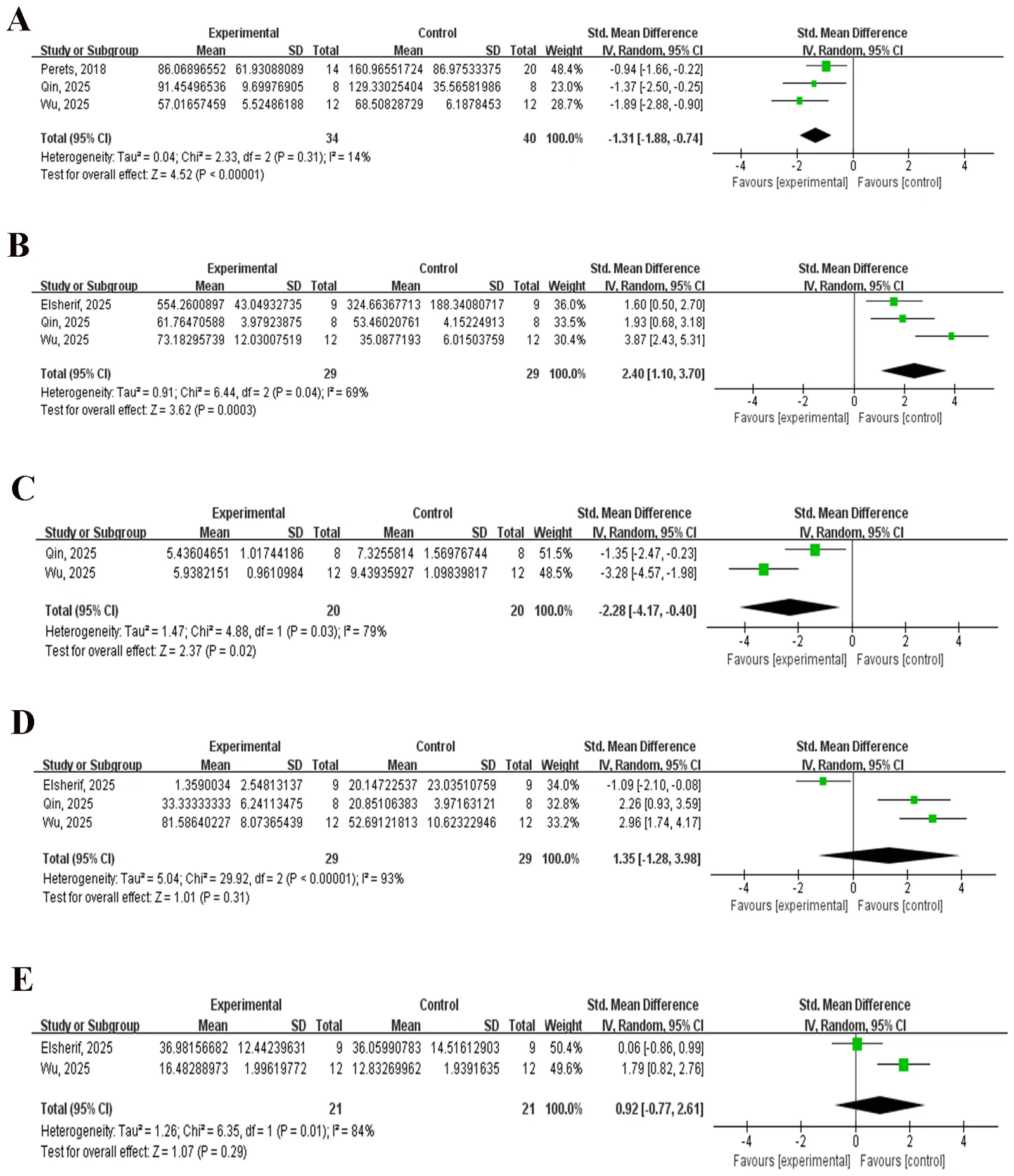
Forest plots demonstrating that stem cell-derived EV treatment ameliorates autistic-like behaviors in ASD animals. **(A)** Self-grooming test. **(B)** Three-chamber social interaction test. **(C)** Marble burying test. **(D)** Open field test. **(E)** Morris Water Maze.

**Table 2:** Summary table of meta-analysis results.

Measures	Studies	Effect estimate	Heterogeneity (I^2^, *P*)
Self-grooming test	3	-1.31 [-1.88, -0.74]	I^2^ = 14%, *P* < 0.001
Three-chamber social interaction test	3	2.40 [1.10, 3.70]	I^2^ = 69%, *P* < 0.001
Marble burying test	2	-2.28 [-4.17, -0.40]	I^2^ = 79%, *P* = 0.02
Open field test	3	1.35 [-1.28, 3.98]	I^2^ = 93%, *P* = 0.31
Morris Water Maze	2	0.92 [-0.77, 2.61]	I^2^ = 84%, *P* = 0.29
IL-6	2	-1.62 [-2.80, -0.44]	I^2^ = 49%, *P* < 0.01
IL-1β	2	-1.67 [-2.65, -0.69]	I^2^ = 27%, *P* < 0.001
TNF-α	2	-1.82 [-4.49, 0.85]	I^2^ = 89%, *P* = 0.18
IL-10	2	1.70 [-0.26, 3.66]	I^2^ = 80%, *P* = 0.09

### 3.5 Stem cell-derived EV treatment modulated inflammatory cytokine levels in ASD animals

Several investigations have evaluated the effects of stem cell-derived EV treatment on inflammatory cytokines in brain tissue of ASD animals. The findings demonstrated that stem cell-derived EV treatment significantly reduced the levels of pro-inflammatory cytokines in brain tissue, including IL-6 (SMD = -1.62, 95% CI: -2.80 to -0.44, I^2^ = 49%, *P* < 0.01) (**Figure 4A**) and IL-1β (SMD = -1.67, 95% CI: -2.65 to -0.69, I^2^ = 27%, *P* < 0.001) (**Figure 4B**). In contrast, stem cell-derived EV treatment did not significantly change the levels of TNF-α (SMD = -1.82, 95% CI: -4.49 to 0.85, I^2^ = 89%, *P* = 0.18) (**Figure 4C**) and IL-10 (SMD = 1.70, 95% CI: -0.26 to 3.66, I^2^ = 80%, *P* = 0.09) (**Figure 4D**) in brain tissue of ASD animals.

**Figure 4:**
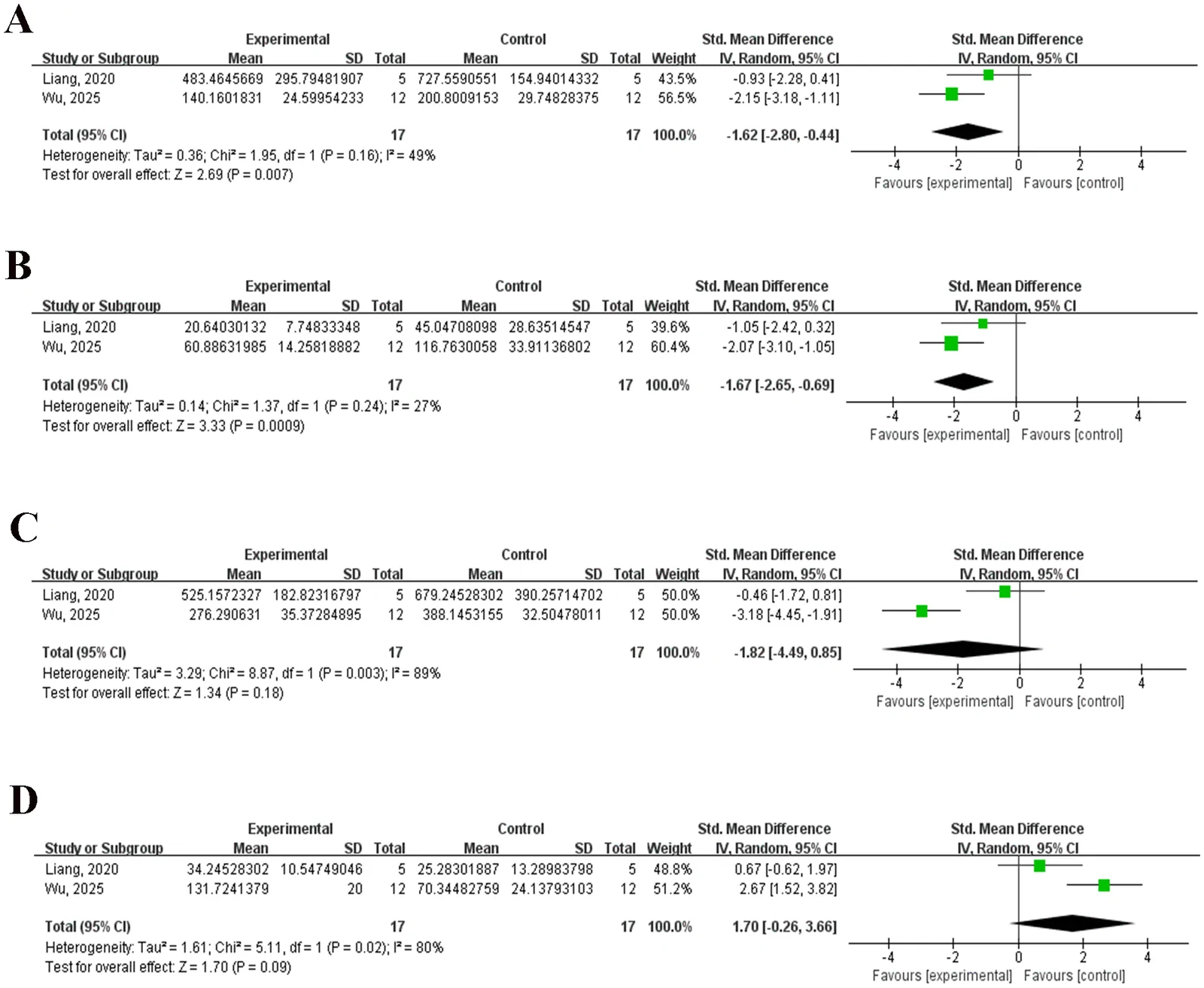
Forest plots illustrating that stem cell-derived EV treatment modulated inflammatory cytokine levels in ASD animals. **(A)** IL-6. **(B)** IL-1β. **(C)** TNF-α. **(D)** IL-10.

## 4 Discussion

The present work represents the first systematic review and meta-analysis investigating the preclinical efficacy of stem cell-derived EVs in animal models of ASD. By pooling data from five studies, we obtained combined effect estimates showing that EV treatment significantly ameliorated the core behaviors of ASD, including self-grooming, sociability deficits in the three-chamber test, and marble-burying behavior. In contrast, pooled analyses revealed no significant effects on open field test or Morris Water Maze. Additionally, EV therapy significantly reduced brain levels of IL-6 and IL-1β, but not TNF-α or IL-10. These data imply that stem cell-derived EVs may alleviate the core symptoms of ASD through IL-6 and IL-1β-driven neuroinflammation, providing a preliminary foundation for further clinical translation.

The three-chamber social interaction test measures sociability (25), while the self-grooming and marble burying tests assess repetitive behaviors (26, 27), which are the core symptoms of ASD. The most compelling conclusion of this meta-analysis is that EV therapy significantly improved these core symptoms. Notably, despite heterogeneity in sources (bone marrow and umbilical cord), administration routes (intravenous injection and nasal administration), and animal models (C57BL/6, BTBR, rats), the meta-analytic estimates remained significant. However, moderate to high heterogeneity was observed for the three-chamber test (I^2^ = 69%) and marble burying test (I^2^ = 79%). We adopted random-effects models to adjust for this heterogeneity, and sensitivity analyses indicated that removal of any one study did not affect the overall effect. This robustness strengthens confidence in our pooled conclusions.

In contrast, the meta-analysis found no significant improvement in the open field test (reflecting locomotor activity and anxiety-like behavior) or the Morris Water Maze (reflecting spatial learning and memory abilities) (28, 29). The small number of studies limited statistical power to detect modest effect sizes. In addition, the high heterogeneity suggests substantial variability in baseline locomotor activity, anxiety levels, or cognitive testing conditions across studies. Furthermore, the lack of effect on open field behavior might imply that EV treatment does not broadly alter general activity and anxiety levels. Similarly, the absence of significant cognitive improvement in the Morris Water Maze might suggest that EV treatment does not robustly affect hippocampal-dependent spatial memory. Larger-scale studies with harmonized protocols are warranted to clarify the observed outcomes.

Regarding neuroinflammation, our pooled estimates reveal that stem cell-derived EVs significantly reduced IL-6 and IL-1β levels in brain tissue. Both cytokines are upstream drivers of neuroinflammation and are implicated in the pathogenesis of ASD (16, 17, 30). Elevated IL-6 and IL-1β can impair synaptic plasticity and contribute to excessive neuronal excitation (31). The heterogeneity of these results is relatively low, supporting the consistency of the anti-inflammatory effects across different studies. In contrast, the meta-analysis did not detect significant changes in TNF-α or the anti-inflammatory cytokine IL-10. The high heterogeneity and wide confidence intervals suggest that the null results may be due to low statistical power and between-study variability. Alternatively, EVs may selectively inhibit specific IL-6 and IL-1β pathways rather than exerting a broad anti-inflammatory effect. Given that the limited number of studies contributed to these cytokine outcomes, no definitive conclusion can be drawn. Future mechanistic studies should directly compare EVs derived from different sources and their impact on specific inflammatory pathways in ASD models.

Beyond pure therapeutic applications, EVs also hold promising diagnostic potential for ASD and exhibit close therapeutic relevance. Circulating EVs can cross the blood-brain barrier and carry molecules from the brain, including microRNAs, proteins, and inflammatory mediators, which can reflect the pathological state of the central nervous system. Studies have shown that exosomal proteins and microRNAs differ in the peripheral blood of ASD patients compared to neurotypical controls (32, 33), suggesting that EVs may become candidate peripheral biomarkers for the diagnosis of ASD. Our previous study reported reduced miR-30b-5p in plasma exosomes and brains of VPA-exposed rats with elevated inflammatory cytokines, while overexpression of miR-30b-5p ameliorated ASD-like behaviors and suppressed inflammation (17), highlighting that EV cargo may function both as a biomarker and a therapeutic target. In addition, evidence suggests that EV-encapsulated molecules can also be used to monitor therapeutic responses (34).

Although preclinical results are positive, there are still many obstacles when translating these findings to humans. Firstly, standards for EVs are not yet fully established. Different laboratories use different methods for isolation and characterization, which affects reproducibility of the research. Secondly, the biological distribution and targeting mechanisms of EVs are not clear. It is currently unknown what fraction of EVs can reach relevant brain regions after nasal or intravenous injection. Thirdly, the long-term safety has not been evaluated, including non-specific effects, immunogenicity, and the impact on neurodevelopment. Fourthly, the animal models have inherent limitations. Animal models cannot reproduce the heterogeneity of human genetic, environmental, and developmental processes. There are significant differences in behavior, cognition, and inflammatory dynamics among different species. Therefore, although this meta-analysis provides a foundation for subsequent research, caution is warranted when applying the evidence to human therapeutics.

Several limitations of this meta-analysis should be noted. First, the pool of studies was small in size (n = 5), with individual meta-analyses relying on only 2–3 studies. Such limited data yield imprecise effect estimates. Heterogeneity metrics such as I^2^ and τ^2^ are unstable and should be interpreted with considerable caution. Treatment effects may also be overestimated due to the limited number of included studies. Therefore, our findings are preliminary and require validation in larger, well-powered preclinical studies. Second, the studies exhibit suboptimal methodological standards. For most studies, the risk of bias was unclear across critical domains, including random sequence generation, allocation concealment, and blinding. This reflects a common defect in preclinical research and may lead to overestimation of treatment effects. Third, there was significant heterogeneity in some indicators, the sources of which could not be fully explored because of the few included studies. Fourth, the follow-up durations were relatively short, and it is still unclear whether the effects of EVs are durable and safe over the long term. Fifth, our meta-analysis only focused on several inflammatory cytokines, while other mechanisms (oxidative stress, mitochondrial function, synaptic plasticity, gut-brain axis) were not investigated.

Based on these limitations, we propose the following suggestions for future preclinical studies to improve the quality of evidence for meta-analyses. First, well-designed experiments following standardized protocols and with large sample sizes should be conducted. Second, standardized procedures for isolating, characterizing, dosing, and administering EVs are urgently needed to improve comparability across studies. Third, the continued efficacy and long-term safety of stem cell-derived EV treatment for ASD must be evaluated through extended follow-up studies. Fourth, the exact mechanisms by which EVs exert effects should be further explored.

## 5 Conclusion

This meta-analysis is the first to comprehensively analyze the preclinical evidence of stem cell-derived EV therapy in ASD. The pooled results show that EV treatment can improve repetitive behaviors and social deficits, with observed associations with reduced IL-6 and IL-1β levels. Stem cell-derived EVs represent a promising approach for ASD treatment, despite the limited number and high heterogeneity of available studies. Our results suggest clinical translation of EV-based therapy for ASD, which will require larger-scale, methodologically rigorous preclinical studies to verify efficacy and safety.

## Data Availability

The original contributions presented in the study are included in the article/**Supplementary Material**. Further inquiries can be directed to the corresponding authors.
